# Alcohol’s Effects on Pair-Bond Maintenance in Male Prairie Voles

**DOI:** 10.3389/fpsyt.2017.00226

**Published:** 2017-11-17

**Authors:** Andre T. Walcott, Andrey E. Ryabinin

**Affiliations:** ^1^Department of Behavioral Neuroscience, Oregon Health and Science University, Portland, OR, United States

**Keywords:** ethanol, alcohol drinking, social attachment, prairie voles (*Microtus ochrogaster*), oxytocin, FosB/ΔFosB, periaqueductal gray

## Abstract

Alcohol abuse can have devastating effects on social relationships. In particular, discrepant patterns of heavy alcohol consumption are associated with increased rates of separation and divorce. Previous studies have attempted to model these effects of alcohol using socially monogamous prairie voles. These studies showed that alcohol consumption can inhibit the formation of pair bonds in this species. While these findings indicated that alcohol’s effects on social attachments can involve biological mechanisms, the formation of pair bonds does not properly model long-term human attachments. To overcome this caveat, this study explored whether discordant or concordant alcohol consumption between individuals within established pairs affects maintenance of pair bonds in male prairie voles. Male and female prairie voles were allowed to form a pair bond for 1 week. Following this 1-week cohabitation period, males received access to 10% continuous ethanol; meanwhile, their female partners had access to either alcohol and water or just water. When there was a discrepancy in alcohol consumption, male prairie voles showed a decrease in partner preference (PP). Conversely, when concordant drinking occurred, males showed no inhibition in PP. Further analysis revealed a decrease in oxytocin immunoreactivity in the paraventricular nucleus of alcohol-exposed males that was independent of the drinking status of their female partners. On the other hand, only discordant alcohol consumption resulted in an increase of FosB immunoreactivity in the periaqueductal gray of male voles, a finding suggesting a potential involvement of this brain region in the effects of alcohol on maintenance of pair bonds. Our studies provide the first evidence that alcohol has effects on established pair bonds and that partner drinking status plays a large role in these effects.

## Introduction

Alcohol is often used as a “social lubricant” to enhance social bonds. On the other hand, alcohol abuse can have detrimental effects on certain social bonds. Among such affected social bonds are long-term relationships between spouses. In a survey exploring the demographic distribution of drinking patterns, 73% of married men and 63% of married women stated that they drink alcohol (1). Heavy alcohol use during a marriage has been associated with decreased marital satisfaction and increased rate of divorce (2–6). In fact, alcohol and drug use is the third most commonly reported reason—behind infidelity and incompatibility—for divorce in the United States (7). However, it has been shown that martial dissatisfaction (8–10) and divorce rates (11–13) tend to increase when there is a discrepancy in husband and wife drinking patterns, but not when the spouses drink in concordance. Given the high prevalence of alcohol consumption in long-term relationships, it is important to understand whether biological mechanisms contribute to the effect of discrepancies in alcohol intake on separation rates.

Using laboratory rodent models can help elucidating the biological mechanisms regulating long-term relationships. One valuable rodent model of mammalian social monogamy is the prairie vole (*Microtus ochrogaster*). Like humans, prairie voles form socially monogamous bonds between same-sex mates (14) and opposite-sex partners (15), and display biparental care for offspring (16, 17). Social attachments, or pair bonds, in prairie voles are mediated by several neurotransmitter and receptor systems that are homologous to those regulating human social affiliations (18–24). These similarities between prairie voles and humans make prairie voles a good translational animal model to study social pair bonds in the laboratory.

Researchers began to investigate the effects of drugs of abuse on pair bonding in prairie voles (25–28). Importantly, not only will prairie voles form social bonds but also they voluntarily self-administer high doses of ethanol without training on a sucrose-fading procedure (29). Unlike most other rodent models, prairie voles can consume higher levels of alcohol when housed in same-sex pairs compared with prairie voles housed in isolation (29). This increase in social drinking in prairie voles is similar to the social facilitation of drinking seen in humans (30). Interestingly, the social facilitation of drinking was previously seen only in same-sex prairie vole pairs, but not between opposite-sex partners (31, 32). Thus, it is clear that different social environments differentially influence the self-administration of alcohol in prairie voles, much like in humans.

Previously, there has not been an adequate amount of research on the effects of alcohol on opposite-sex pair bonds in rodent models. To the best of our knowledge, only one study has investigated these effects. Anacker et al. (33) explored the effects of alcohol on the formation of pair bonds in male and female prairie voles. Briefly, male and female prairie voles were paired for 24 h while simultaneously receiving access to 10% ethanol and water or only water. To determine their pair-bond strength, animals were then tested in the partner preference test (PPT). Males exposed to alcohol showed no preference for their partner when compared with the control group. By contrast, females showed facilitation in the preference for their partner compared with the control group. The opposite effects of alcohol consumption on the formation of partner preference (PP) in males versus females were accompanied by sex-specific effects of alcohol on neural activity in several brain regions. These findings demonstrated that alcohol’s effects on social pair bonds could have biological underpinnings. However, alcohol’s effects on pair-bond formation do not fully model the disruption that the discrepancy in alcohol intake has on long-term relationships.

In this study, we used male prairie voles to investigate the effects of discrepancies in alcohol intake on established pair bonds. We hypothesized that when there was a discrepancy in alcohol access between partners, the prairie voles would show a decrease in PP compared with voles that had a partner who was given access to alcohol. Our results demonstrate that discordant, but not concordant, alcohol drinking leads to a decrease in PP in male prairie voles. Follow-up experiments testing effects of alcohol on immunoreactivity of oxytocin, arginine vasopressin (AVP), and FosB suggest that the effect of discrepant drinking may involve activation of the periaqueductal gray (PAG). To the best of our knowledge, this is the first demonstration of alcohol’s effects on pair-bond maintenance and the first investigation of neurocircuits that might mediate this effect.

## Materials and Methods

### Animals

Adult male and female prairie voles (*n* = 150; 76–126 days old) from our breeding colony at the VA Portland Health Care System (VAPORHCS) Veterinary Medical Unit were used in these experiments. All animals were weaned at 21 days and housed in same-sex sibling groups (two to four animals per cage) in cages (27 cm × 27 cm × 13 cm) under a 14:10 light/dark cycle, until the start of experiments. All subjects had access to cotton nestlets and *ad libitum* access to water and a diet of mixed rabbit chow (LabDiet Hi-Fiber Rabbit; PMI Nutrition International, Richmond, IN, USA), corn (Nutrena Cleaned Grains; Cargill, Inc., Minneapolis, MN, USA), and oats (Grainland Select Grains; Grainland Cooperative, Eureka, IL, USA) throughout the entire experiment. All experiments were conducted in accordance with the Institutional Animal Care and Use Committees at the VAPORHCS and Oregon Health & Science University, Portland, OR, USA.

### Housing Conditions

At the start of experiments, male subjects were placed in a standard plastic housing cage with a female partner for 1 week to establish a pair bond. The following week, all subjects and opposite-sex partners were placed in a mesh-divided social housing cage (27 cm × 27 cm × 13 cm). These social housing cages have been described previously (29, 34). Briefly, they contain a mesh divider in the middle of the cage to separate each animal in the pair. These cages prevent animals from mating, but allow olfactory and visual social contact with partners to still occur. These cages also allow the monitoring of individual fluid consumption. It has previously been described that these mesh-divided social housing cages do not affect established pair bonds (35).

### Two-Bottle Choice Test

During the period when animals were housed in mesh-divided cages, all animals were given continuous access to two 25 mL glass cylinders fitted with a metal sipper tube and rubber stopper. Three experimental conditions were used in these set of experiments: (1) both male and female partners were given access to one bottle of water and a second bottle containing 10% ethanol (Both EtOH); (2) the male was given access to one bottle of water and a second bottle of 10% ethanol, whereas the female partner was given access to two bottles of water (Male only EtOH); and (3) both male and female partners were given access to two bottles of water (Control). Bottles were monitored every 24 h, and then bottles were refilled and their position was switched to prevent side bias.

Average daily alcohol consumption for each prairie vole was calculated by dividing the grams of alcohol by the kilogram of body weight. Alcohol consumption and preference for alcohol were both analyzed by repeated-measures ANOVA for the effects of days, treatment, and their interaction after testing for normality using the Shapiro–Wilk test (all data sets for alcohol consumption and preference passed the normality test; *p* > 0.05). Significance for all experiments was set at *p* < 0.05.

### Partner Preference Test

Partner preference test was used as a standard way to test pair bonding in prairie voles (36, 37). Immediately following the two-bottle choice paradigm (described earlier), the effect of discordant and concordant alcohol consumption on pair-bond maintenance in male subjects (total *n* = 23) was assessed using a 3-h PPT. The PPT was performed in a three-chambered apparatus with the partner stimulus (*n* = 23) tethered in one chamber, the female stranger (*n* = 23) tethered in the opposite chamber, and the subject animal placed in a center, non-social chamber and allowed to move freely throughout the three chambers. The female stranger animals were housed in mesh-divided cages with siblings and were exposed to the same experimental treatment as the female partners. PPT was videotaped and was viewed later for behavioral analysis.

The main outcome of PPT is the duration of time the male subject spends huddling with either the partner or stranger animal; this is a measure of social preference. An experimenter who was blinded to group assignment and trained in detecting huddling behavior used VLC Media Player (Boston, MA, USA) to view the recorded videos. Behavior Tracker 1.0 software was used to measure the amount of time each animal spent huddling with the partner or stranger at a 5× playback speed. Male huddling time with female partners was analyzed using the Brown–Forsythe test to determine normality. Partner huddling was normally distributed (*F*_2,15_ = 0.532, *p* = 0.598), thus the PPT data met the assumptions required to use parametric test for analysis. PPT data were analyzed by two-way ANOVA with stimulus animal (i.e., partner or stranger) and treatment (i.e., alcohol or water access) as between-subjects factors and followed by a Fisher’s LSD *post hoc* test.

### Resident-Intruder (RI) Test

Another way to measure pair-bond maintenance is through the RI test. Previously, it has been described that attack frequency toward a same-sex stranger during the RI test can be used to measure the strength of a pair bond (18, 38, 39). Therefore, a different set of male animals (total *n* = 27) from the ones described earlier was used for the RI test. These animals were exposed to the two-bottle choice paradigm and the mesh-divided housing as above, but instead of the PPT; they were put through the RI test. The 10-min RI test took place in the mesh-divided cages (on the subject’s side) immediately following the voluntary alcohol intake procedure. The female partner (*n* = 27) was removed from her side and placed in a separate holding cage during the test. The male strangers (*n* = 27) were housed in mesh-divided cages with siblings and were exposed to the same experimental treatment as the male subjects. The RI test was videotaped and was viewed later for behavioral analysis.

The main outcome of the RI test is the frequency of aggressive interactions (lunges, bites, chases, and offensive rears) toward the stranger male. An observer blind to experimental conditions used VLC Media Player (Boston, MA, USA) to view the recorded videos. JWatcher behavioral observation software (V 1.0, Macquarie University and UCLA) was used to measure the frequency of aggressive interactions at a 1× playback speed. To determine if we could use a parametric test to analyze the RI data, we used the Brown–Forsythe test to analyze normality. The Brown–Forsythe test revealed that the RI data were normally disturbed (*F*_2,15_ = 2.647, *p* = 0.104), thus a parametric test was used. RI data were analyzed by one-way ANOVA to determine the effects concordant and discordant alcohol drinking had on aggressive frequency.

### Embryo Analysis

After the PPT and RI tests, female partners were euthanized. Embryos were then removed and weighed. The average weights of all apparent embryos in an animal were used for analysis. Embryo weights were analyzed because the stage of pregnancy is positively correlated with measurements of maintenance of pair bonds (35). Specifically, male prairie voles that have a female partner that had been pregnant for 10 days or more spend significantly more time huddling with their partner over a stranger, compared with males who have a female partner who had been pregnant for less than 10 days. Average embryo weights that correspond to >0.3 g are considered to be optimal impregnation (greater or equal to 10 days pregnant at the time of testing), while weights <0.3 g are considered suboptimal impregnation (less than 10 days pregnant at the time of testing) (35, 38). Five female partners in the PPT experiment and nine female partners in the RI experiment had suboptimal pregnancies. As a result, in the final analysis, there were six animals per group in the PPT experiment and five to seven animals per group in the RI analysis. Only data from male subjects that had a female partner, which reached optimal pregnancy, were used in statistical analysis within this study.

### Immunohistochemistry

To determine the potential molecular mechanisms involved in effects of discordant and concordant alcohol consumption on established pair bonds, subjects (*n* = 5–7 per group) from the RI experiment were euthanized by CO_2_ immediately after the completion of the RI test. Brains were then extracted, fixed in 2% paraformaldehyde/PBS for 24 h, and cryoprotected using 20% and then 30% sucrose/PBS. Brain tissue was sliced at 40-μm coronal sections and stored in 0.1% sodium azide until IHC assay. Sections containing 18 brain regions were selected for analysis. Regions of interest were determined by using the Paxinos and Franklin (40) mouse brain atlas. The following primary antibodies were used: anti-oxytocin (1:20,000, Peninsula Laboratories), anti-AVP (1:50,000, Peninsula Laboratories), and anti-FosB (1:27,000, Abcam). An anti-rabbit secondary antibody made in goat (Vector Laboratory, Inc.) was used, and then signal was amplified using a Vectastain ABC kit (Vector Laboratory, Inc.). Tissue was then stained using a metal enhanced diaminobenzidine substrate kit (Thermo Fisher Scientific) and visualized using a Leica DM4000 bright-field microscope. All cells that were stained above background were counted using automatic cell counting techniques by ImageJ. An experimenter blinded to the condition of the subjects analyzed the data by one-way ANOVA. Significant effects were followed up by a Fisher’s LSD *post hoc* test.

## Results

### Effects of Concordant and Discordant Drinking on Maintenance of PP

To compare effects of concordant and discordant drinking on pair-bond maintenance, we examined three groups of adult male prairie voles. *Control* males were cohabitated with females for 2 weeks. During the second week, a mesh divider was introduced between the male and the female allowing the experimenter to monitor fluid consumption of each member of the pair. Males of the *Both EtOH* group were housed similarly, but during the second week both the male and the female were introduced to a choice between two fluids: water and 10% ethanol. Since both males and females in these pairs were exposed to alcohol, they were considered to experience concordant drinking. Males of the *Male only EtOH* group were also cohabitated for 2 weeks, but only male animals had access to a choice between water and 10% ethanol during the second week. Therefore, these males experienced discordant drinking.

**Figure 1:**
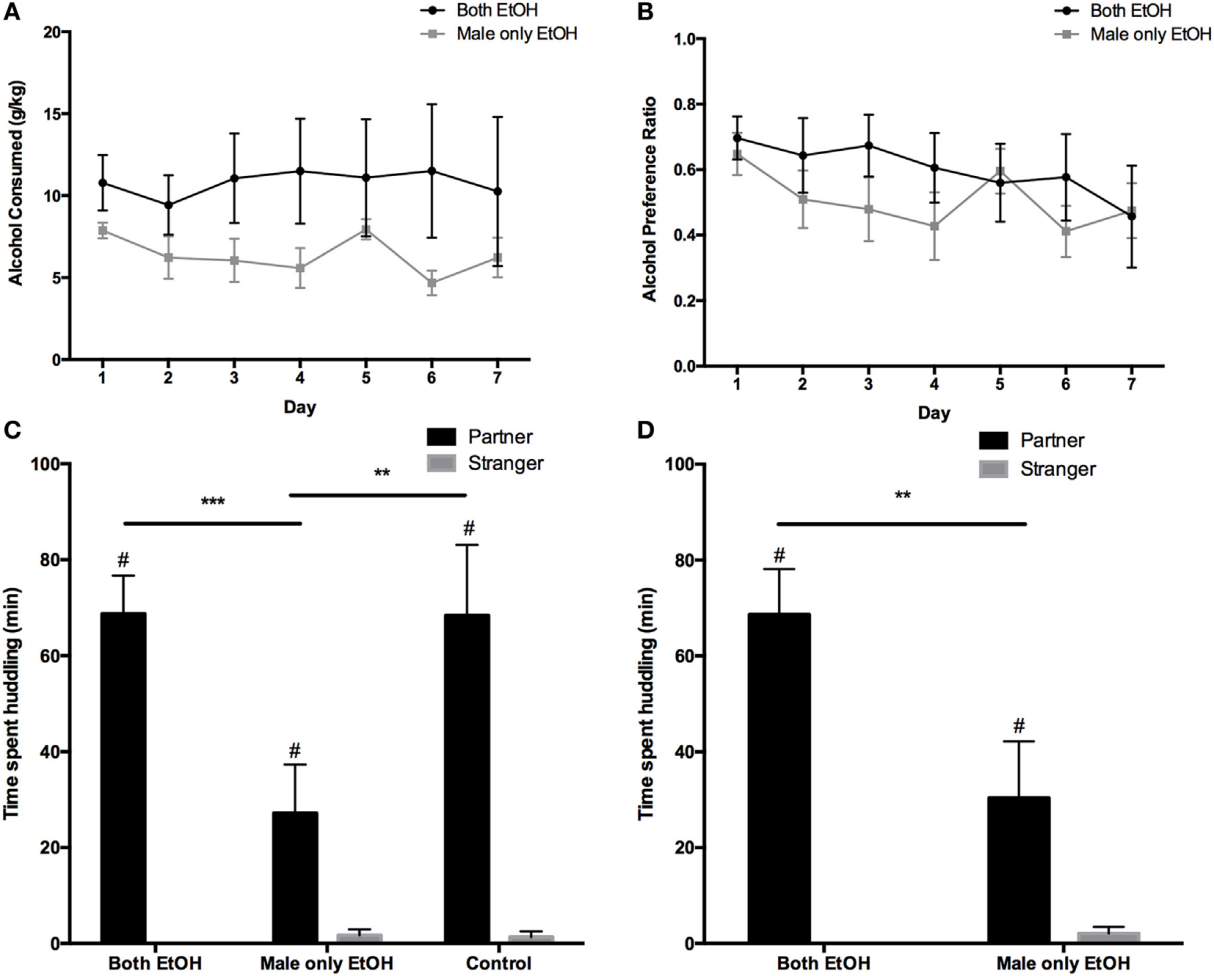
The effects of discordant and concordant alcohol consumption on the partner preference test (PPT) in male voles. **(A)** Males that had a female partner that was exposed to alcohol showed an increase in alcohol consumption during the two-choice test, but showed no difference in **(B)** alcohol preference. **(C)** Males showed a partner preference (PP) under all three experimental conditions, but PP was significantly decreased when only the male had access to EtOH compared with when both animals were exposed to EtOH or only water. **(D)** When the EtoH exposed groups were matched for alcohol consumption and preference, there was still a significant decrease in the amount of time the males in the Male only EtOH group spent huddling with their partners, compared with the males in the Both EtOH group. *p < 0.05; **p < 0.01; ***p < 0.001; ^#^significant effect of animal (*p* ≤ 0.04). Error bars indicate mean ± SEM.

When both male and female partners were given access to alcohol, males consumed on average 10.8 ± 0.3 (mean ± SEM) grams of alcohol per kilogram of body weight (g/kg) per day over a 7-day drinking period. Meanwhile, when only the male was given access to alcohol, males consumed on average 6.4 ± 0.5 g/kg of 10% ethanol over the same 7-day period. Depending on day and animal, alcohol consumption ranged from 0.0 to 30.8 g/kg and 0.9 to 11.9 g/kg in the Both EtOH and Male only EtOH groups, respectively. Analysis of the alcohol consumption revealed that males in the Both EtOH group significantly increased the amount of 10% ethanol consumed compared with males in the Male only EtOH group (*F*_1,70_ = 11.820, *p* = 0.001; Figure 1A). There was no significant difference between the amount of 10% ethanol consumed each day (*F*_6,70_ = 0.135, *p* = 0.991) and no significant interaction between day and treatment group (*F*_6,70_ = 0.199, *p* = 0.976). Alcohol preference was not significantly different between the males in the Both EtOH (range: 0.0–98.8%) group and the males in the Male only EtOH (range: 4.9–85.2%) group (*F*_1,70_ = 3.127, *p* = 0.081; Figure 1B). There was no significant difference in alcohol preference between each day (*F*_6,70_ = 0.921, *p* = 0.485) and no significant interaction between day and treatment group (*F*_6,70_ = 0.451, *p* = 0.842).

In addition to the analysis of alcohol intake and preference, water intake was analyzed. There was no significant difference in water intake between males in the Both EtOH group and males in the Male only EtOH group (*F*_1,70_ = 0.016, *p* = 0.900). There was no significant difference between the amount of water consumed each day (*F*_6,70_ = 1.165, *p* = 0.335), and no interaction between day and treatment group (*F*_6,70_ = 0.378, *p* = 0.891).

Next, we tested the effects of discordant and concordant drinking on PP in male prairie voles. During the PPT, there was a significant effect of stimulus animal (partner versus stranger) on huddling time (*F*_1,30_ = 67.70, *p* < 0.0001), a significant effect of treatment (Both EtOH versus Male only EtOH versus Control) on huddling time (*F*_2,30_ = 4.236, *p* = 0.002), and a significant interaction between treatment and stimulus animal (*F*_2,30_ = 4.701, *p* = 0.017; Figure 1C). *Post hoc* analysis revealed that males in all three groups spent significantly more time huddling with their partner compared with the stranger animal (*p* < 0.05). The most important finding was that males in the Both EtOH (*p* = 0.0009) and Control (*p* = 0.001) groups spent significantly more time huddling with their partners compared with the amount of time the males spent huddling with their partner in the Male only EtOH group.

To determine if the difference in alcohol consumption between the Both EtOH and Male only EtOH groups contributed to the difference in PP between these groups, we matched groups for alcohol consumption by eliminating data from three animals. This manipulation eliminated the significant difference in alcohol intake between the Both EtOH and Male only EtOH groups (*p* = 0.237). Reanalysis of the PPT in animals with matched intakes confirmed the significant effect of stimulus animal on huddling (*F*_1,14_ = 37.610, *p* < 0.0001), a significant effect of treatment on huddling time (*F*_1,14_ = 5.237, *p* = 0.038), and a significant interaction between stimulus animal and treatment (*F*_1,14_ = 6.514, *p* = 0.023; Figure 1D). *Post hoc* analysis revealed that both groups had a significant PP (*p* < 0.05) and again showed a significant increase in the amount of the time males in the Both EtOH group spent huddling with their partner compared with the males in the Male only EtOH group. This finding confirmed that the difference in the amount of time the males spent huddling with their partners was not attributed to the difference in alcohol consumption in the Both EtOH and Male only EtOH groups.

### Effects of Concordant and Discordant Drinking on Selective Aggression

When sexually naïve prairie voles are introduced to a novel conspecific they tend to show affiliative behaviors (41). These affiliative behaviors start to become directed specifically toward their partner once they formed a pair bond (18, 38, 42). In addition to more affiliative behaviors toward their partner, pair-bonded voles also display more aggressive behaviors toward unfamiliar same-sex stimulus animals. Therefore, we explored if males in the Male only EtOH group would show a change in the amount of aggressive behaviors toward an unfamiliar same-sex stimulus animal during the RI test. A separate group of animals were cohoused for a week and then introduced to the mesh divider cages with each animal receiving the two-bottle choice paradigm, as described earlier.

**Figure 2:**
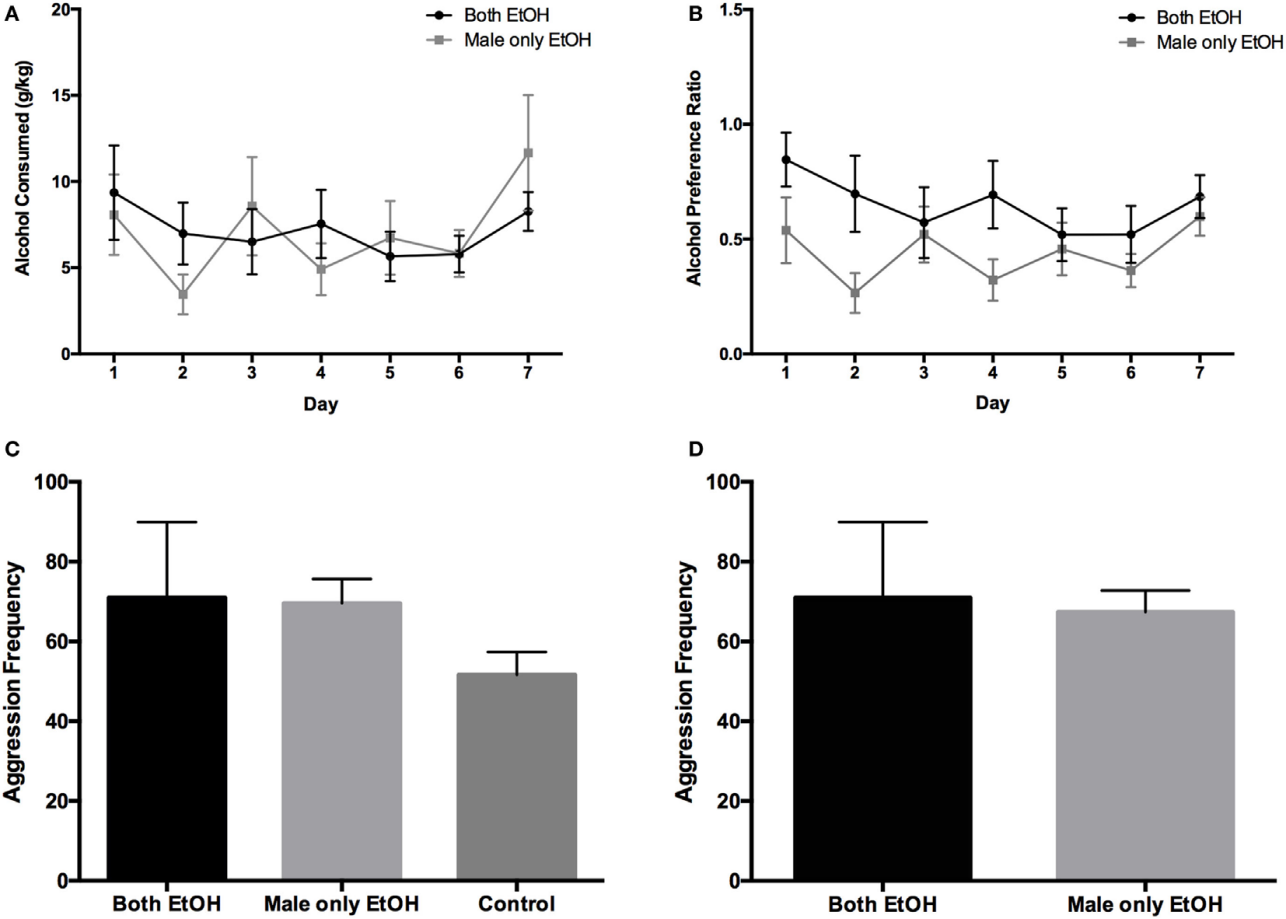
The effects of discordant and concordant alcohol consumption on aggression frequency during the resident-intruder (RI) test in male prairie voles. **(A)** Alcohol consumption in males did not differ between treatment groups. **(B)** There was a significant difference between the alcohol preference ratio between the Both EtOH and Male only EtOH groups. **(C)** Aggression frequency toward a stranger male during the RI test did not differ between male subjects in the three treatment groups. **(D)** When the alcohol consumption and alcohol preference ratio between the two alcohol-consuming groups were matched, there was still no difference in aggression frequency between the two groups. Error bars indicate mean ± SEM.

When both partners were given access to 10% ethanol, males consumed on average 7.2 ± 0.5 g/kg (range: 0.7–17.0 g/kg) during the 7-day drinking period. When only the male was given access to 10% ethanol, males consumed on average 7.0 ± 1.0 g/kg (range: 0.1–15.0 g/kg). Analysis of alcohol consumption revealed that there was no statistical difference in the amount of alcohol consumed by the males in the Both EtOH and the Male only EtOH groups (*F*_1,70_ = 0.012, *p* = 0.915; Figure 2A). There was no significant difference between the amount of 10% ethanol consumed each day (*F*_6,70_ = 1.264, *p* = 0.285) and no significant interaction between day and treatment group (*F*_6,70_ = 0.674, *p* = 0.671). Males in the Both EtOH group showed a 64 ± 4.4% preference (range: 6.0–100%) for alcohol, whereas the males in the Male only EtOH group showed a 44 ± 4.7% preference (range: 2.7–91.2%) for alcohol; thus, leading to the occurrence of a significant difference between the two groups (*F*_1,70_ = 11.10, *p* = 0.001; Figure 2B). There was no significant difference in alcohol preference between each day (*F*_6,70_ = 1.204, *p* = 0.315) and no significant interaction between day and treatment group (*F*_6,70_ = 0.895, *p* = 0.504).

To complement the alcohol intake and preference data, we analyzed the amount of water intake between groups. There was a significant difference between the amount of water consumed between the males in the Both EtOH group and males in the Male only EtOH group (*F*_1,70_ = 17.050, *p* < 0.0001). There was no significant difference in water intake between days (*F*_6,70_ = 1.519, *p* = 0.185) and no significant interaction between day and treatment group (*F*_6,70_ = 0.935, *p* = 0.475).

To determine if discordant alcohol consumption between partners contributes to a change in aggressive behavior, we ran the RI test after seven days of the two-bottle choice paradigm. We found no significant difference in the number of aggressive behaviors toward the unfamiliar same-sex intruder between the three treatment groups (*F*_2,15_ = 1.066, *p* = 0.369; Figure 2C). To determine if the difference in alcohol preference ratio contributed to the non-significant effect of aggressive behaviors, we matched groups for alcohol consumption and preference and reanalyzed the RI test for the Both EtOH and Male only EtOH groups. Similarly to the previous results, we found no difference in the number of aggressive behaviors toward the RI (*t*_9_ = 0.183, *p* = 0.859; Figure 2D).

### Immunohistochemical Analysis of Potential Substrates of Alcohol’s Effects on Pair-Bond Maintenance

**Figure 3:**
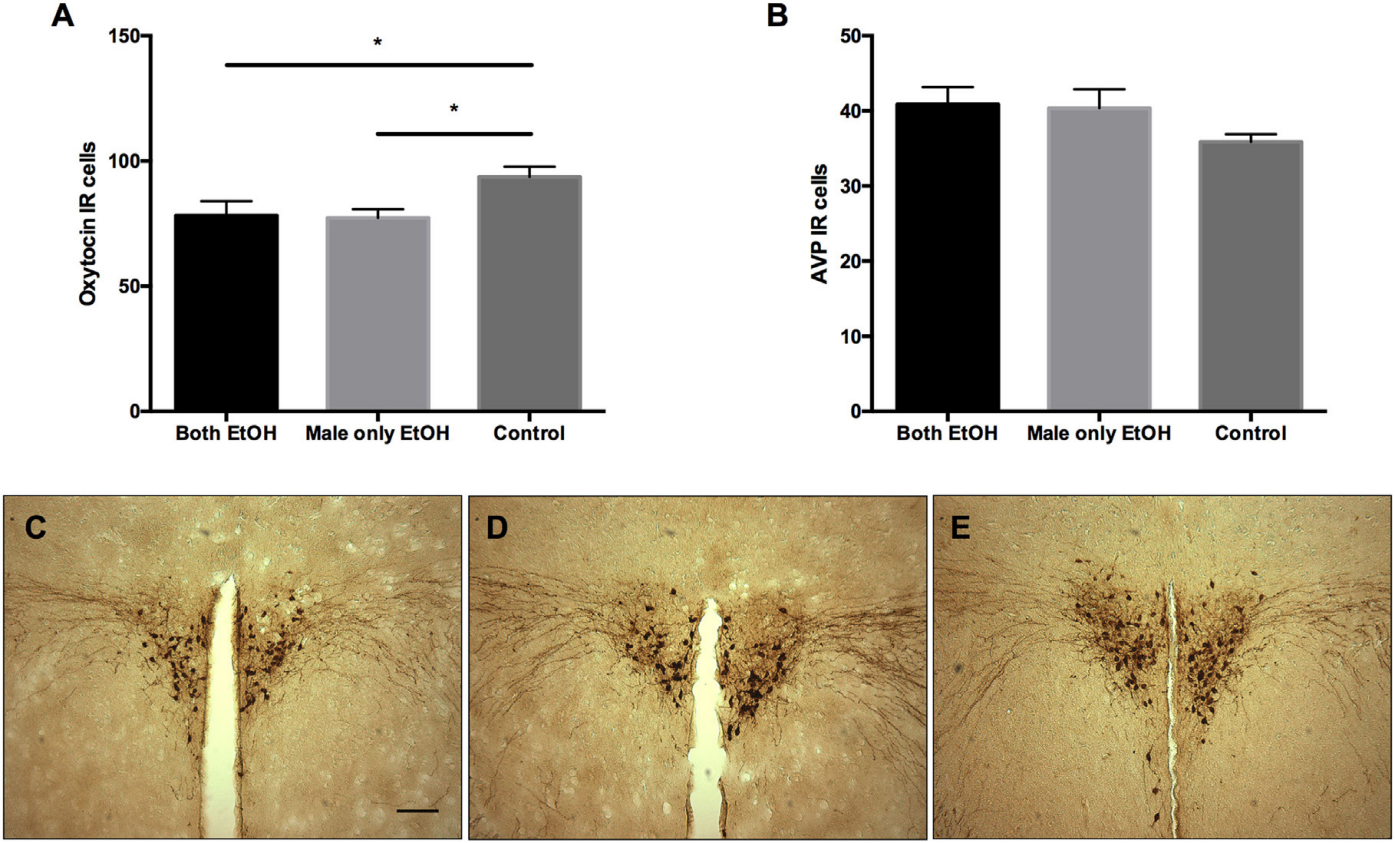
Immunoreactivity for oxytocin and AVP in the PVN. **(A)** Number of oxytocin-immunnoreactive cells within the paraventricular nucleus of the hypothalamus (PVN) is significantly higher in the Control group compared with the Both EtOH and Male only EtOH groups. **(B)** Number of AVP-immunoreactive cells within the PVN does not differ between the three treatment groups. Representative photomicrographs of oxytocin immunoreactivity in the PVN in the **(C)** Both EtOH (scale bar, 0.2 μm), **(D)** Male only EtOH, and **(E)** Control groups. *p < 0.05. Error bars indicate mean ± SEM.

Oxytocin and AVP play important roles in pair bonding, and oxytocin levels in the neurons of the paraventricular nucleus of hypothalamus (PVN) have been shown to decrease following long-term alcohol consumption (43). Therefore, we tested whether the effects of alcohol on PP in the experiment above could be due to changes in the levels of these peptides. Immediately following the RI test, animals were euthanized and brains were cryopreserved for immunohistochemistry. We found a significant effect of treatment for oxytocin-ir in the PVN (*F*_2,17_ = 3.753, *p* = 0.045; Figure 3A). *Post hoc* analysis revealed that the males that were given access to alcohol had a significant decrease of the amount of oxytocin-ir cells within the PVN (*p* < 0.05). Photomicrographs of oxytocin-ir in the PVN are shown for all three groups in Figures 3C–E. By contrast, we found no significant difference between the number of AVP-ir cells within the PVN between the three groups (*F*_2,17_ = 1.576, *p* = 0.236; Figure 3B).

Although we identified an effect of alcohol consumption on oxytocin levels, these levels were not different between the Both EtOH and Male only EtOH groups. Therefore, an effect of alcohol on PVN oxytocin levels could not completely explain the difference in PP between these groups, suggesting that other systems are involved in the effects of discordant drinking on pair-bond maintenance.

**Figure 4:**
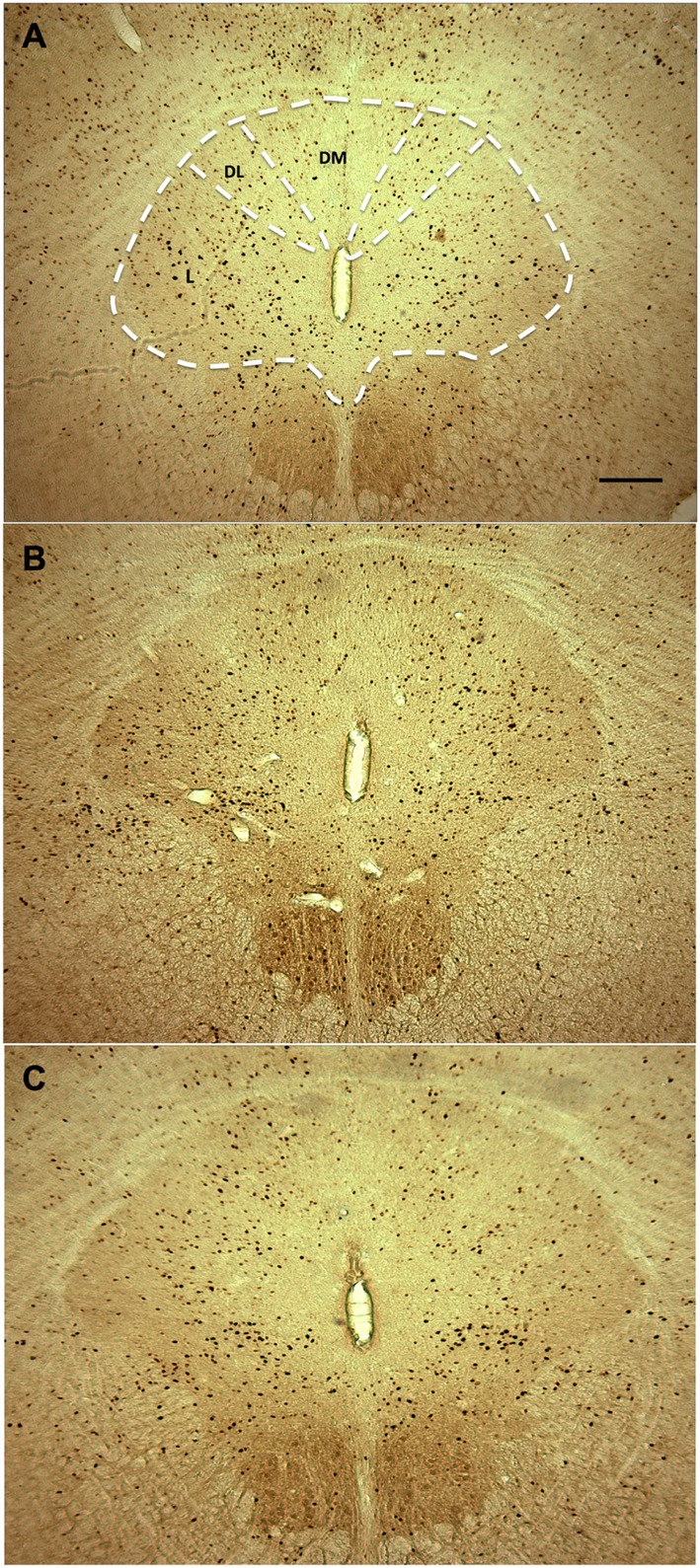
Representative photomicrographs for FosB immunoreactivity in the subregions of the periaqueductal gray (PAG) in **(A)** Both EtOH, **(B)** Male only EtOH, and **(C)** H_2_O. DM, dorsal medial PAG; DL, dorsal lateral PAG; L, lateral PAG.

**Figure 5:**
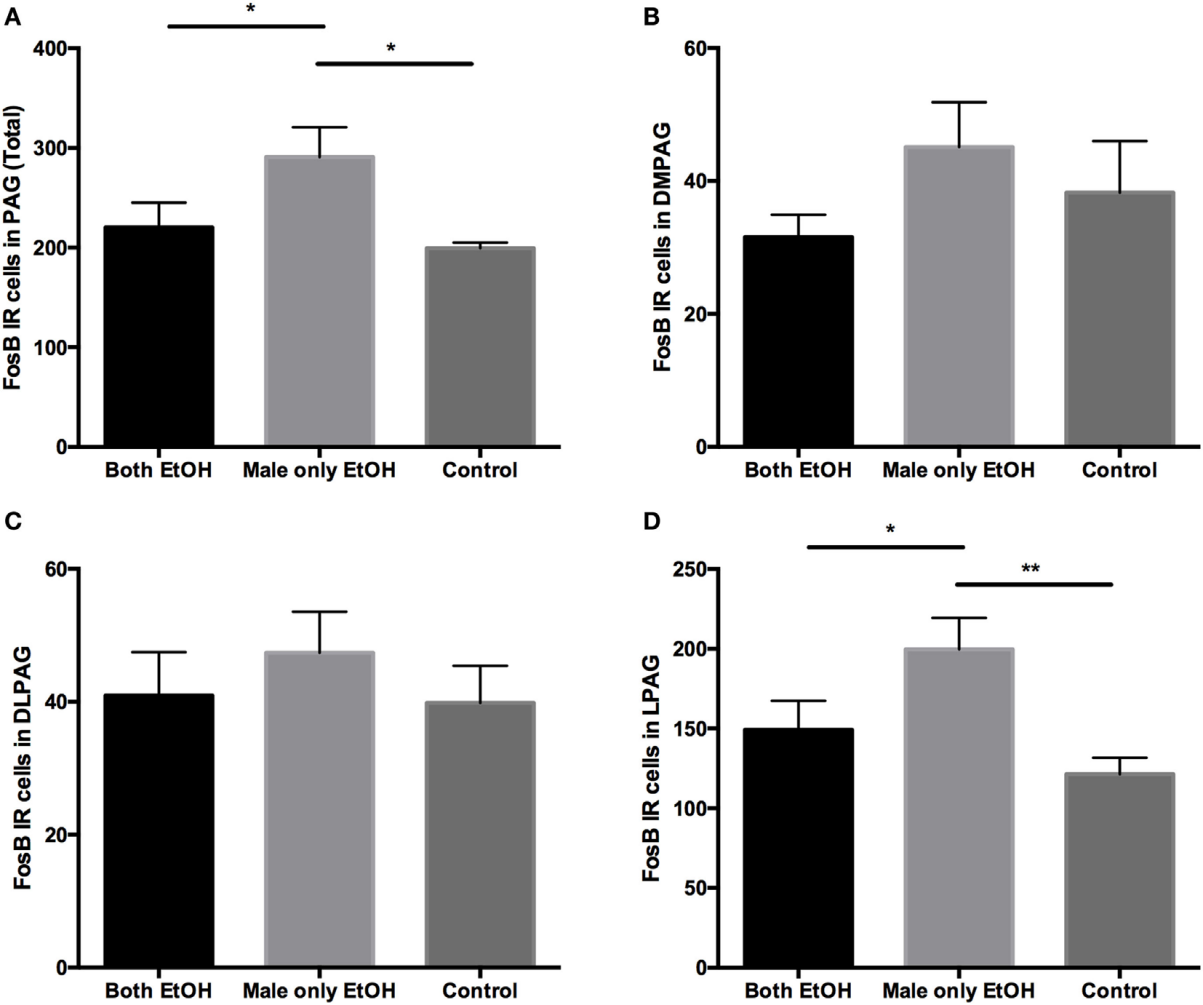
Immunoreactivity for FosB in the subregions of the periaqueductal gray (PAG). **(A)** The number of immunoreactivity FosB cells within the PAG was significantly increased in the Male only EtOH group compared with the Both EtOH and Control groups. There was no difference in the number of FosB cells in the PAG when both partners were exposed to EtOH compared with when both partners were exposed to only water. The PAG was divided into three subregions: **(B)** dorsal medial, **(C)** dorsal lateral, and **(D)** lateral. The three different two-bottle choice conditions had no significant effect on the number of FosB cells within the dorsal medial and dorsal lateral regions of the PAG. The number of FosB cells within the lateral region of the PAG significantly differed between the three treatment groups **(D)**. The Male only EtOH group showed an increase in the number of FosB cells within the lateral PAG when compared with the Both EtOH and Control groups, thus leading to an increase in the total number of FosB cells within the PAG. *p < 0.05; **p < 0.01. Error bars indicate mean ± SEM.

**Figure 6:**
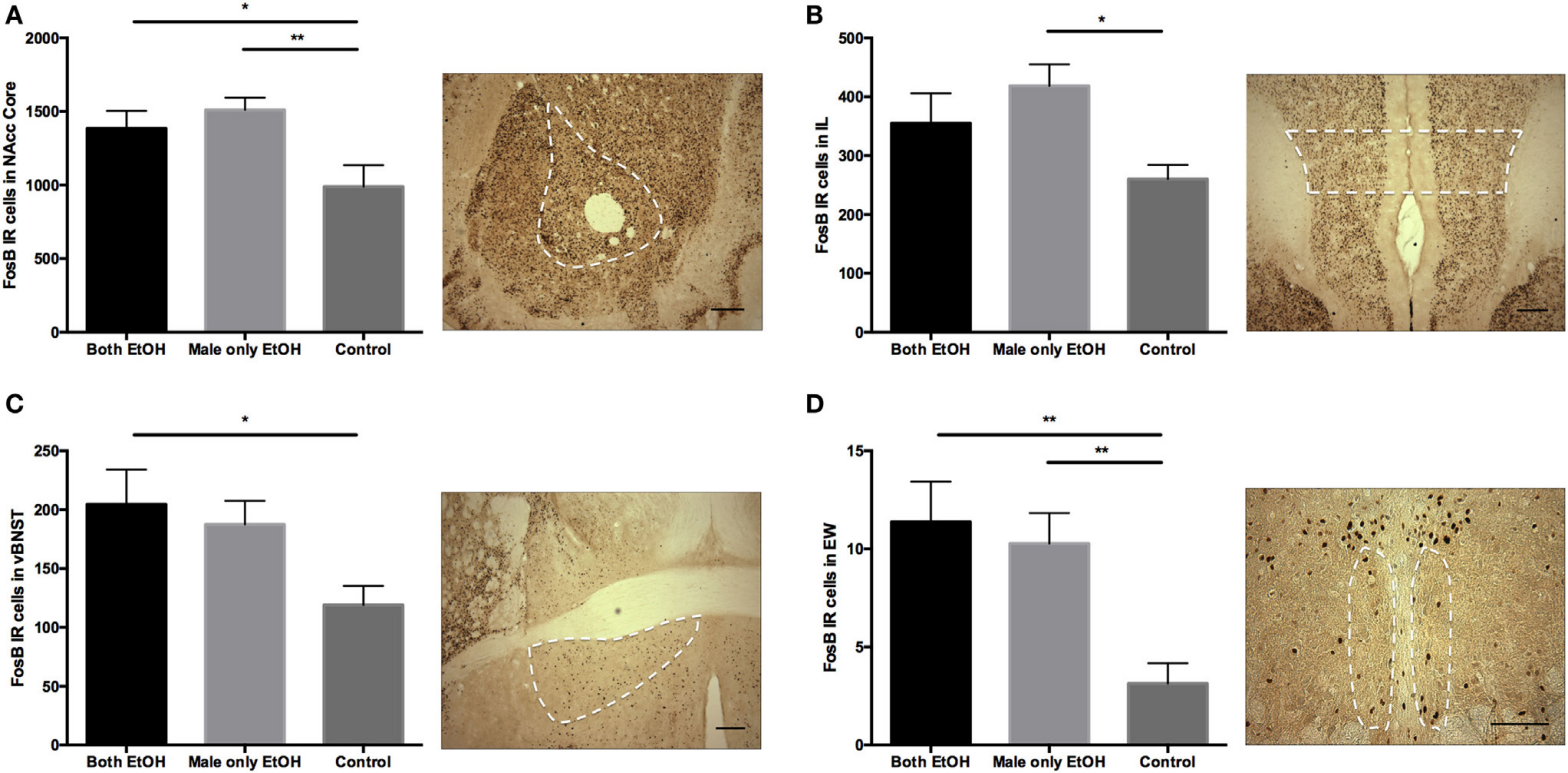
FosB immunoreactivity enhanced in regions within the Both EtOH and Male only EtOH groups. The number of FosB positive cells in four additional brain regions showed a significant difference between the three groups. **(A)** Number of FosB positive cells within the nucleus accumbens core (NAcc Core) was significantly increased within the Both EtOH and Male only EtOH groups compared with the Control group. **(B)** Number of FosB positive cells in the infralimbic cortex (IL) was significantly increased in the Male only EtOH group compared with the Control group. **(C)** Subjects in the Both EtOH group had an increase in the number of FosB positive cells in the ventral bed nucleus of the stria terminalis (vBNST) compared with the Control group. **(D)** The number of FosB positive cells in the centrally projecting Edinger–Westphal nucleus (EW) was significantly increased in the Both EtOH and Male only EtOH groups compared with the Control group. *p < 0.05; **p < 0.01. Error bars indicated mean ± SEM.

To begin identifying other neural substrates potentially involved effects of discordant drinking on PP, we examined FosB-ir across 18 different brain regions in the slices collected in the experiment above (Table 1). Five of the 18 brain regions showed significant differences between groups (Table 1). The number of positive FosB-ir cells within the PAG was significantly different between the three treatment groups. Specifically, males in the Male only EtOH group had an increase in FosB-ir cells in the entire PAG compared with the males in the Both EtOH and Control groups (Figures 4 and 5A). In addition to the effects in the PAG, there were significant between group differences in the nucleus accumbens core (NAcc Core) (*F*_2,17_ = 5.227, *p* = 0.017; Figure 6A), infralimbic cortex (IL) (*F*_2,17_ = 3.808, *p* = 0.043; Figure 6B), ventral bed nucleus of the stria terminalis (vBNST) (*F*_2,17_ = 3.607, *p* = 0.05; Figure 6C), and centrally projecting Edinger–Westphal nucleus (EW) (*F*_2,17_ = 6.931, *p* = 0.006; Figure 6D). In all four of these regions, *post hoc* analysis revealed that FosB-ir in the males in the Both EtOH and Male only EtOH groups was not significantly different from each other. However, FosB-ir in Both EtOH or Male only EtOH groups was significantly different from males in the Control group. Thus, of all the brain regions examined, only PAG showed patterns of FosB expression different between males exhibiting discordant versus concordant drinking. To investigate if the difference was caused by a global increase in FosB-ir cells in the entire PAG or its particular subregion, we subdivided the PAG into three regions: dorsal medial (DMPAG), dorsal lateral (DLPAG), and lateral (LPAG) (Figure 4). There were no between group differences in the number of FosB-ir cells in the DMPAG (*F*_2,17_ = 0.297, *p* = 0.297) and DLPAG (*F*_2,17_ = 0.441, *p* = 0.65). However, there was a between group difference in the LPAG (*F*_2,17_ = 5.311, *p* = 0.016; Figures 5B–D). *Post hoc* analysis revealed that males in the Male only EtOH group had a significant increase in the number of FosB-ir cells in the LPAG compared with the males in the Both EtOH (*p* < 0.05) and Control (*p* < 0.01) groups.

**Table 1:** The mean ± SEM for positive FosB cells for each experimental group per brain region examined.

Brain region	Both EtOH	Male only EtOH	Control	*p*-Value
Anterior cingulate (CG1)	781.3 ± 37.0	701.4 ± 75.5	775.9 ± 36.8	0.299
Anterior cingulate (CG2)	911.8 ± 95.3	919.0 ± 103.2	888.7 ± 38.7	0.968
Agranular insula	250.9 ± 30.4	237.9 ± 22.3	254.5 ± 16.7	0.881
Granular insula	378.2 ± 50.9	362.1 ± 35.7	353.7 ± 16.9	0.904
Infralimbic cortex	354.9 ± 51.0	418.8 ± 36.4	260.2 ± 24.0	**0.043**
Retrosplenial cortex	1,395.0 ± 154.9	1,333.0 ± 79.2	1,202.0 ± 45.40	0.470
Dorsal lateral striatum	953.9 ± 92.7	868.6 ± 149.3	1,007.0 ± 79.7	0.700
Dorsal medial striatum	1,383.0 ± 89.5	1,380.0 ± 104.3	1,287.0 ± 95.1	0.741
Nucleus accumbens core	1,385.0 ± 119.0	1,510 ± 84.0	988.6 ± 145.6	**0.017**
Nucleus accumbens shell	945.6 ± 86.0	1,021.0 ± 91.8	821.1 ± 129.0	0.405
Lateral septum	384.7 ± 35.0	372.2 ± 32.6	328.6 ± 26.7	0.467
Dorsal bed nucleus of the stria terminalis	197.8 ± 33.1	165.1 ± 25.8	112.5 ± 12.6	0.106
Ventral bed nucleus of the stria terminalis	204.6 ± 29.6	187.6 ± 19.9	119.1 ± 16.1	**0.050**
Paraventricular of the hypothalamus	27.3 ± 5.3	35.9 ± 5.0	47.9 ± 16.7	0.209
Hippocampus (CA1–3)	198.5 ± 49.1	308.1 ± 53.0	322.9 ± 59.5	0.248
Dentate gyrus	592.6 ± 120.3	747.8 ± 124.0	478.5 ± 107.1	0.307
Periaqueductal gray (PAG) (total)	220.3 ± 24.8	290.8 ± 30.1	199.4 ± 5.6	**0.037**
PAG (dorsal medial)	31.6 ± 3.4	45.1 ± 6.8	38.3 ± 7.8	0.297
PAG (dorsal lateral)	40.9 ± 6.6	47.4 ± 6.2	39.83 ± 5.6	0.650
PAG (lateral)	149.2 ± 18.1	199.7 ± 19.7	121.3 ± 10.3	**0.016**
Edinger–Westphal nucleus	11.4 ± 2.1	10.3 ± 1.6	3.1 ± 1.0	**0.006**

*The *p* value from the ANOVA for each brain region is listed in column 5. Regions with significant *p* values (α ≤ 0.05) are in bold text*.

## Discussion

This study investigated how discordant and concordant alcohol drinking influences established pair bonds in male prairie voles. We found that male prairie voles had a decreased PP if the drinking was discordant, but not when it was concordant. Specifically, PP was decreased when the males were drinking alcohol, while their female partner was drinking only water. By contrast, when both male and female partners were drinking alcohol, male prairie voles showed no reduction in PP compared with when both partners were exposed to only water. Interestingly, when males were tested for selective aggression we saw no group differences between the amount of aggressive behaviors displayed toward an unfamiliar same-sex prairie vole in the RI test. Previous studies have shown that drugs of abuse administered during the formation of a pair bond can affect PP (25, 26, 33, 44). To the best of our knowledge, this is the first study to demonstrate an effect of a drug of abuse on PP when given after a pair bond has been formed.

We chose to investigate the effects of alcohol on maintenance of PP using the voluntary two-bottle choice drinking procedure because voluntary and involuntary modes of drug administration in rodent models engage different neurocircuits (45–48). This procedure allowed us also to investigate whether female partners would influence alcohol consumption in the males. We found that female drinking status had an inconsistent tendency to influence alcohol self-administration in male prairie voles. Thus, in the first experiment the amount of alcohol consumed was significantly higher in the Both EtOH versus the Male only EtOH group. While this difference was not significant in the second experiment, males in the Both EtOH group showed a significantly higher preference for alcohol than males in the Male only EtOH group. Previous research has shown that same-sex prairie voles will socially facilitate the amount of alcohol each partner consumes (31), but opposite-sex partners do not significantly influence drinking behaviors (32). The latter study had methodological differences in relation to this study, including that males were gonadectomized and partners were exposed to increasing alcohol concentration (3–10%) over a 12-day period. It is possible that these two methodological differences were the reason why we saw that females can influence males’ self-administration in this study, but not in previous studies. It is also possible that significant effects of alcohol intake would be reached if more animals were used in this study. Importantly for the main result of the current investigation, when the Both EtOH and Male only EtOH groups were matched for alcohol intake and preference, only the Male only EtOH group showed decreased PP. This finding indicated that discordant drinking, but not concordant drinking inhibits maintenance of the pair bond in prairie voles.

Remarkably, the inhibitory effects of discordant drinking on pair-bond maintenance observed here parallel epidemiological data on the association between alcohol consumption and marital dissolution in humans. Thus, couples with high alcohol drinking in both spouses are often found to be as stable as abstinent couples and significantly more stable than couples in which only one spouse drinks (12, 13, 49). This observation appears to be very consistent when it is based on the number of separations, and less so when it is based on subjective measures of marital satisfaction (6, 50–52). Interestingly, the effect of such discordant drug taking on marital stability is relatively specific for alcohol, as it is not observed in relations to smoking and marijuana (11).

Importantly, a recent study by Leonard et al. (11) suggests that effects of discordant drinking on divorce rates could be stronger in heavy drinking wives than in heavy drinking husbands. Specifically, the effect of discordant drinking in husbands was statistically significant when data were unadjusted for sociodemographic, antisocial personality, and depression. When these three factors were adjusted for, heavy drinkers-husband heavy couples displayed a trend for the increase in divorce rates compared with non-using couples. By contrast, the discrepant heavy drinkers-wife heavy group in their study showed a significant increase in divorce rates compared with non-using couples when adjusted or unadjusted for the same factors. Our study for the first time analyzed effects of discordant drinking on pair-bond maintenance in voles and initially focused on males. Our future experiments will address how a discrepancy in alcohol consumption affects pair-bond maintenance in female prairie voles. In addition, it will be important to investigate whether a different duration of alcohol access or withdrawal (versus intoxication) could modulate the effects of alcohol on pair-bond maintenance. Nevertheless, the current findings of disruptive effects of discordant alcohol drinking during 1 week on pair-bond maintenance in male voles provide evidence that such effects have biological underpinnings. Therefore, prairie voles can be used to investigate neural substrates of the effects of alcohol use and abuse on social monogamous behaviors. Such investigations were initiated in this study.

Our analysis on PVN showed that alcohol drinking in prairie voles leads to a decrease in the number of oxytocin-immunoreactive cells within the PVN. This reduction in oxytocin-immunoreactive cells occurred in both alcohol-consuming groups, regardless of female drinking status. This alcohol-mediated decrease in oxytocin levels is in agreement with two previous studies. Silva et al. (53) found that rats that received an alcohol solution as their only liquid source for 6 or 10 months showed a decrease in the amount of oxytocin-immunoreactive and AVP-expressing cells in the PVN, which was attributable to cell death. Interestingly, the surviving cells showed hypertrophy, such that oxytocin mRNA and AVP mRNA levels per cell compensated for the cell loss. A more recent study performed by Stevenson et al. (43) in prairie voles showed that 7 weeks of voluntary alcohol consumption of 15% ethanol in a two-bottle choice procedure resulted in a decrease in the number of oxytocin cells in the PVN of male animals. As in our experiments, there was no significant reduction in the number of PVN AVP neurons. These findings suggest that while a prolonged exposure to alcohol can affect the AVP system, the PVN oxytocin neurons are sensitive to relatively short exposures. In this study, the decrease in the number of oxytocin neurons was observed after an even shorter (1 week) exposure to alcohol than in the Stevenson study. While Stevenson et al. (43) and our study did not specifically address whether the reduction in oxytocin-positive neurons is attributable to cell death, the rapid effect observed in our study suggests an effect on oxytocin expression, rather than loss of specific neurons.

Our observation that only 1 week of voluntary alcohol consumption was required for the significant reduction in oxytocin neurons indicates high sensitivity of this system to alcohol. On the other hand, the fact that both concordant and discordant drinking affected the PVN oxytocin neurons suggests that effects of alcohol on this system alone cannot explain the selective effect of discordant drinking on maintenance of PP. Therefore, additional mechanisms involved in this selective effect need to be explored.

We began searching for such involved additional systems by testing levels of FosB immunoreactivity in 18 brain regions that could be potentially involved in regulation of social attachment or effects of alcohol. FosB is an immediate early gene. Expression of immediate early genes Fos, FosB, and JunB can be used to map acute changes in neural activity (54–56). However, repeated exposure to the same stimulus can attenuate the immediate early gene response in neurons (57, 58). This decreased sensitivity to repeated treatment makes mapping changes in neural activity following 1 week of continuous exposure to alcohol difficult. In contrast to other immediate early genes, FosB also encodes a short deltaFosB protein, which gradually accumulates with repeated treatments (59–62). The anti-FosB antibody used in the current experiments recognizes both the full-length FosB protein and deltaFosB. Therefore, our FosB immunohistochemistry was capable of detecting effects of both acute and prolonged effects of alcohol. We have initiated mapping effects of prolonged alcohol consumption on neural activity in our earlier studies in mice (63, 64).

In this study, we detected five brain regions in which the number of FosB-immunoreactive cells was regulated by alcohol. We observed that alcohol, but not the drinking status of the female partner, increased FosB in NAcc Core, IL, vBNST, and EW. NAcc and EW have been previously repeatedly found to respond with immediate early gene induction to either involuntary or voluntary alcohol exposure (63–68). IL and BNST have been found to respond with induction of the immediate early gene c-fos to involuntary alcohol exposure (69, 70). Importantly in relation to behavior results, we also found that males, who are in the discordant drinking group, have an increase in the amount of FosB-immunoreactive cells in the PAG compared with the males in the concordant and control group. Specifically, the LPAG was driving the increase in FosB immunoreactivity for the entire PAG region. This result suggests that LPAG could be involved in mediating selective effects of discordant drinking on maintenance of PP.

While previous research has suggested that PAG is involved in social behaviors, most studies focused on its activity in response to a social stress (71, 72). Related to affiliative behaviors, PAG activation was been shown to be associated with exposure to maternal emotional responses in humans (73). In agreement with this idea, Miranda-Paiva et al. (74) showed that injections of the opioid antagonist naloxone into the rostral lateral PAG reversed inhibitory effects of morphine on maternal behaviors in rats. While the neurocircuitry of pair-bond formation has been elucidated, only a few studies to date have examined the potential contribution of specific brain regions in maintenance of pair bonds. Bales et al. (75) and Maninger et al. (76) have mapped changes in glucose metabolism following pair bonding in monogamous titi monkeys. They found significant increases in glucose metabolism in several brain regions, but not in the PAG. Resendez et al. (39) have shown that manipulations of the opioid system in the nucleus accumbens regulated maintenance of pair bonds in prairie voles. The potential causal contribution of PAG to maintenance or formation of pair bonds has not been tested. Such studies will need to be performed in the future.

Taken together, we have identified that discordant, but not concordant, voluntary alcohol consumption inhibits maintenance of pair bonds in male prairie voles, as evidenced by decreased PP. This effect is reminiscent of effects of heavy discordant drinking on marital dissolution in humans. We have identified potential neural substrates involved in these effects. Future studies should use comprehensive pharmacological and molecular approaches testing whether these inhibitory effects of discordant drinking can be reversed or prevented.

## Ethics Statement

All experiments were conducted in accordance with the Institutional Animal Care and Use Committees (IACUC) at the VA Portland Health Care System (VAPORHCS) and Oregon Health & Science University (OHSU), Portland, OR, USA. The protocol was approved by the VAPORHCS IACUC.

## Author Contributions

AW and AR have designed the study and written the manuscript. AW has performed all experiments.

## Conflict of Interest Statement

The authors declare that the research was conducted in the absence of any commercial or financial relationships that could be construed as a potential conflict of interest.
